# Growth from the Ciliary Ligament

**Published:** 1831

**Authors:** 


### XIII.

### GROWTH FROM THE CILIARY LIGAMENT.

A CHILD, æt. 10, was struck upon the eye with the closed hand, without occasioning any evident inflammation afterwards. In about a week a small reddish tumour formed in the anterior chamber at the anterior side of the eye, appearing to be attached only to the ciliary ligament. It soon became as large as half a small pea, was convex anteriorly, extended nearly across the pupil, the circularity of which remained, occasioned no pain nor inflammation, and neither impeded the motions of the iris, nor changed its colour. Under the influence of mercury the tumour gradually became absorbed, and never again returned. Did the tumour consist of organized lymph?—*ibid.*

